# Thousands of missed genes found in bacterial genomes and their analysis with COMBREX

**DOI:** 10.1186/1745-6150-7-37

**Published:** 2012-10-30

**Authors:** Derrick E Wood, Henry Lin, Ami Levy-Moonshine, Rajiswari Swaminathan, Yi-Chien Chang, Brian P Anton, Lais Osmani, Martin Steffen, Simon Kasif, Steven L Salzberg

**Affiliations:** 1 Department of Computer Science, University of Maryland, College Park, MD, 20742, USA; 2 Center for Bioinformatics and Computational Biology, University of Maryland, College Park, MD, 20742, USA; 3 McKusick-Nathans Institute of Genetic Medicine, Johns Hopkins University School of Medicine, Baltimore, MD, 21205, USA; 4 Department of Biomedical Engineering, Boston University, Boston, MA, 02215, USA; 5 Bioinformatics Program, Boston University, Boston, MA, 02215, USA; 6 New England Biolabs, 240 County Road, Ipswich, MA, 01938, USA; 7 Department of Pathology and Laboratory Medicine, Boston University School of Medicine, Boston University, Boston, MA, 02218, USA; 8 Department of Biostatistics, Bloomberg School of Public Health, Johns Hopkins University, Baltimore, MD, 21205, USA

## Abstract

**Background:**

The dramatic reduction in the cost of sequencing has allowed many researchers to join in the effort of sequencing and annotating prokaryotic genomes. Annotation methods vary considerably and may fail to identify some genes. Here we draw attention to a large number of likely genes missing from annotations using common tools such as Glimmer and BLAST.

**Results:**

By analyzing 1,474 prokaryotic genome annotations in GenBank, we identify 13,602 likely missed genes that are homologs to non-hypothetical proteins, and 11,792 likely missed genes that are homologs only to hypothetical proteins, yet have supporting evidence of their protein-coding nature from COMBREX, a newly created gene function database. We also estimate the likelihood that each potential missing gene found is a genuine protein-coding gene using COMBREX.

**Conclusions:**

Our analysis of the causes of missed genes suggests that larger annotation centers tend to produce annotations with fewer missed genes than smaller centers, and many of the missed genes are short genes <300 bp. Over 1,000 of the likely missed genes could be associated with phenotype information available in COMBREX. 359 of these genes, found in pathogenic organisms, may be potential targets for pharmaceutical research. The newly identified genes are available on COMBREX’s website.

**Reviewers:**

This article was reviewed by Daniel Haft, Arcady Mushegian, and M. Pilar Francino (nominated by David Ardell).

## Background

Bacterial gene identification has improved considerably since the first bacterial genome, *Haemophilus influenzae* Rd KW20, was sequenced in 1995 [1]. Bacterial genome annotation is usually done through an automated process that identifies most genes very accurately, with sensitivity sometimes exceeding 99% [2]. As a result of the declining cost and increasing ease of genome sequencing in recent years, 1,699 prokaryotic genomes (113 archaea and 1,586 bacteria) have been completely sequenced, and nearly 5,000 more draft genomes have been deposited in public archives. The genomes of most of these species have been sequenced and annotated by relatively large sequencing centers, but many smaller centers and even individual laboratories have contributed some genomes as well. The continuing reduction in the cost of sequencing suggests that the trend towards sequencing by small laboratories will increase substantially in the future. Many of these smaller laboratories do not have substantial in-house bioinformatics expertise. The annotation process can vary greatly from one center to the next, and even within a center it varies from year to year, with different programs used for gene finding, alignment, and assigning gene names.

Several different gene finding programs have been used over the years, including Glimmer [2-4], GeneMark [5,6], and others, and each of these programs has itself gone through different versions that produced changes (mostly improvements) in their performance. The one program that has been consistently used over the years and across all species is BLAST [7], which is most commonly used to search predicted protein-coding genes against an ever-growing database of previously reported proteins. Significant sequence similarity with a protein in another species is strong evidence that the predicted protein is genuine, especially if the target species is evolutionarily distant. BLAST searches for homologous sequences have long been the gold standard of evidence for gene prediction.

As a result of the various methodologies, annotation is not very consistent between prokaryotic genomes, even for different strains of the same species, unless the annotation was all performed at the same sequencing center and within a relatively short time period. Genes can be missing from the annotation of some strains and present in others; their start codon positions can vary widely, yielding genes with apparently different lengths; genes can be labeled as pseudogenes or not, depending on the conventions used by the original annotators; and genes can be annotated on the wrong strand or the wrong reading frame. Other differences in annotation programs or in the parameters used to run them can also influence the set of genes found, for example by omitting genes below an arbitrary length threshold. Addressing these inconsistencies should improve current methodologies for prokaryotic annotation.

Obtaining perfect annotation for a bacterial genome is still beyond our reach, even though methods continue to improve. Thus it is reasonable to assume that there are genes missing in current annotations. Finding even some of those genes that have been omitted and correcting other flaws will have direct effects on our biological understanding of the species in question. Many of the missed genes can be associated with specific biochemical functions, thus contributing to our knowledge of the species’ molecular machinery. In some cases, for example when a missed gene in a potentially pathogenic organism is associated with antibiotic resistance, identifying it can help us better understand the causes and treatments of infections.

For this reason, we took a very broad look at all the completely sequenced prokaryotic genomes to determine how many likely genes are simply missing from the annotation and are easily found with our proposed pipeline. We focused on this question because protein sequence homology, as measured by BLAST, provides a highly reliable, consistent tool for identifying missing genes. Although gene identification is generally much better for prokaryotes than for eukaryotes (whose gene structure is much more complex), many genes are nonetheless missing entirely from finished, published genomes.

In this work, we do not intend to find the entire set of missing genes but instead to demonstrate a relatively simple way to find a large set of likely missed genes. In addition to identifying thousands of missing genes, we also provide some possible explanations for their omission, and provide analysis and information about each missed gene in a publicly available database (see details below). It is important to note that there are additional issues with gene annotation that are beyond the scope of our work, but should be addressed by the annotation community. In addition to missing genes, we found many other inconsistencies, including genes of varying lengths and with clearly incompatible names, but resolving those inconsistencies is much more difficult, often involving manual curation.

Another problem that may arise in genome annotation is the problem of overannotation, the incorrect annotation of ORFs in genomes which are not true genes. Unfortunately, there are no reliable methods for definitively determining that a particular ORF is not a true gene. Experiments can show that an ORF is not expressed under certain conditions, but showing that an ORF is not expressed under all conditions is not feasible. There have been attempts to quantify overannotation by examining the length distribution of annotated genes, and showing that the length distribution of all annotated genes does not match the length distribution of only annotated genes with supporting homology to known genes [8]. Since it is impossible to precisely determine the extent of overannotation in a genome, we focused on finding unannotated genes that had considerable computational support for their protein-coding nature and were able to be found with freely available and commonly used software.

Our approach to finding missed genes involves using a combination of Glimmer to find ORFs that were likely to be protein-coding genes, which were not present in the existing annotations from GenBank [9], and then using BLAST to find genes that had significant sequence similarity to previously annotated genes. After ruling out potential pseudogenes, we were left with 52,605 ORFs, which we called *candidate missed genes*, from 1,474 completely sequenced prokaryotic genome annotations. Although many of the candidate missed genes had sequence similarity to proteins with functional annotations, providing strong evidence that they were true missed genes, there were also many candidate missed genes with sequence similarity to only hypothetical proteins, which are putative proteins with no known function. These hypothetical proteins may have only been predicted by gene prediction programs without additional evidence to support a claim that they are true genes. For those candidate missed genes with homology only to hypothetical proteins, we needed additional information to determine if they were indeed genes.

To this end, we make use of the newly available resource COMBREX [10], which is an online database [11] containing functional predictions and phenotype information for more than 3 million microbial genes (see Methods for more details). Using this knowledgebase, we were able to assign each gene to a COMBREX support level, which helps estimate how likely each potential missed gene found is to be a true protein-coding gene. For instance, if a candidate missed gene has a homolog in COMBREX that has been experimentally cloned and tested for function, we assign the gene to the strong COMBREX support level, and our confidence that this candidate missed gene is a fully functional, protein-coding gene increases. In our analysis, most of the candidate missed genes that share sequence similarity with non-hypothetical proteins were found to have strong COMBREX support. For the candidate missed genes that only have sequence similarity to hypothetical proteins, a significant number were also found to have evidence from COMBREX showing that they are likely protein-coding genes. Additionally, we were able to use COMBREX to assign functional and phenotype information to many of the missed genes we identified.

Previously, Warren, et al. [12] performed a similar study to find missing genes in 1,297 annotated prokaryotic chromosomes and plasmids in RefSeq. They reported over 38,000 of what they termed “absent annotations”, or “putative genes by similarity to currently annotated genes” [12]. Our criteria for determining candidate missed genes roughly match their criteria for determining “absent annotations,” although we find more candidate missed genes, as we analyze more genomes. Warren, et al. also found 1,000 additional “missed genes,” which are unannotated ORFs with sequence similarity to other ORFs in other distant species.

Our analysis differs from Warren, et al. by going further to determine the subset of candidate missed genes that have strong support to be actual protein-coding genes, and analyzing them with COMBREX. We use COMBREX both to provide further evidence of the gene’s protein-coding nature, and to draw attention to candidate missed genes with important phenotypes that might be of special interest. We also suggest possible reasons for the omission of missed genes, which can be put into practice to improve future annotation efforts.

Although the analysis of Warren, et al. may find more missed genes, as they analyze every intergenic ORF, our focus on predicted yet unannotated genes is able to find a comparable number of missed genes while expending less computational effort in the task of searching for homologous genes. We found that Glimmer predicted over 97% of genes in RefSeq bacterial genome annotations, suggesting that our approach will find a substantial number of missed genes without needing to search all intergenic ORFs. Even though a small number of missed genes may be overlooked by our approach, our goal with this study was not to find all missed genes, but rather a large subset thereof that could be easily found through existing tools. This large subset of missed genes, while not complete, should nonetheless be useful for the research community.

Currently, the entire set of missed genes is accessible online [13] in the form of downloadable lists of genes and sequences, divided by some basic criteria. Eventually, these genes will be fully integrated into COMBREX, which will allow one to search for a particular gene based on specific attributes, view information associated with the gene, and utilize other functionality available from COMBREX.

## Methods

### Overview of analysis

**Figure 1:**
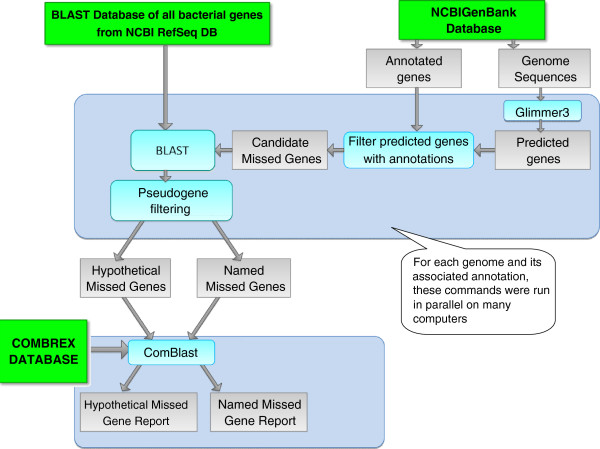
**Data flow through our analysis pipeline.** Annotations and sequences were obtained from GenBank, and all sequences were processed with the Glimmer 3 gene finder to obtain gene predictions. Sets of predicted genes were filtered to exclude annotated genes and pseudogenes to obtain a set of candidate missed genes. These predicted genes were input as queries to BLAST against a database of all bacterial genes in RefSeq. Predicted genes were then designated as named missed genes or hypothetical missed genes, based on if they had a significant alignment to a non-hypothetical protein, or only aligned to hypothetical proteins, respectively. Each of these two sets were further analyzed by ComBlast, which uses BLAST and the COMBREX database to associate genes with additional attributes, such as experimentally determined function, 3D structure, conservation and phenotype information and assign a COMBREX support level to each potential missed gene.

The process used to identify missed genes is summarized in Figure 1. We began by looking at a set of 1,574 prokaryotic chromosomes with GenBank annotations from 1,474 completely sequenced genomes. We used Glimmer3 [2] and a few consecutive filtering steps to identify a set of *candidate missed genes*. These genes were further separated into two distinct subsets based on the nature of the homologs of each individual candidate missed gene. A candidate missed gene that shared significant sequence similarity (based on BLAST similarity scores) with a known gene with non-hypothetical annotation was termed a *named missed gene*. Named missed genes are very likely to be missed genes, given their homology to a known protein with functional annotation. The remaining candidate missed genes were termed *hypothetical missed genes*. These two phases of the pipeline are described in more detail in the first part of this Methods section.

In the next step, we mined COMBREX for functional information about the candidate missed genes. We were able to assign functional and phenotype information to many of the missed genes using ComBlast, a tool that associates the query missed genes with existing data in COMBREX through sequence similarity methods. Based on the data stored in COMBREX, we were also able to assign COMBREX support levels to each missed gene, indicating whether or not it was a true protein-coding gene. Both COMBREX and its use in our analysis are described later in this section.

### Preprocessing and filtering

As shown in Figure 1, we first downloaded all GenBank files (files with a .gbk extension) corresponding to prokaryotic genomes from NCBI [9] on May 16, 2011. We then removed any GenBank files that represented plasmids so that we could focus on the main chromosomes. We decided to exclude plasmids from our analysis because gene prediction methods do not work well on very small sequences, and plasmids typically represent only a small percentage of a genome. We also removed 39 GenBank files that did not contain any annotations at all, presumably because the annotations have not yet been completed. This was an important filtering step as it eliminated around 100,000 of the missing genes we originally thought we found.

Similarly, we took care to remove any draft genomes, as Glimmer may make gene predictions which extend beyond the end of a contig or extend into a region containing many ambiguous nucleotides in the sequence. Since we were unsure if the new genes we found for these genomes were true missed genes, we decided instead to exclude draft and incomplete sequences that we had initially found in the list of “complete” genomes at NCBI. We excluded 36 genomes containing the phrases “draft” or “nearly complete” in the header line, or whose sequences contain more than 10 distinct locations with at least 5 consecutive ambiguous nucleotides. After performing these initial filtering steps, we were left with 1,574 GenBank files, for which we used Glimmer3 and BLAST to find our final set of candidate missed genes.

### Gene prediction and finding missed genes

For each of the 1,574 chromosomes, we generated a set of *ab initio* gene predictions by running Glimmer3 with the g3-iterated.csh script, yielding roughly 4.94 million predicted genes. The only modifications we made to the standard options in the g3-iterated.csh script were for 35 chromosomes, mostly from the genus *Mycoplasma*, that use a non-standard genetic code, where we ran Glimmer3 with the option "-z 4" to set the stop codons used by Glimmer3 to be only TAG and TAA.

We then compared the predicted genes with the annotations in the original GenBank file to find roughly 350,000 predicted genes missed in the GenBank annotation. A predicted gene was considered present in the original annotation if its 3' end was shared with any annotated CDS in the GenBank file. Predicted genes were also eliminated if they overlapped other annotated features, such as RNAs, gene features without a CDS, and CDS entries with a '/pseudo’ tag, by more than 50%.

For the roughly 350,000 gene predictions that remained, to determine which of these might be true missed genes, we created a BLAST database containing all proteins from every bacterial genome listed in the RefSeq database [14], which was approximately 4.2 million proteins as of March 28, 2011. We then translated and aligned each of the remaining predicted genes against this protein database.

Although the use of homology searches will yield many useful results, some of the false predicted genes may also have a low-scoring alignment that, absent any further processing, would lead us to call this false gene a missed gene. Therefore, to ensure that the pairs of homologous genes implied by these BLAST alignments were indicative of true pairs (as opposed to alignments that could occur simply by random chance), we used three filters: an E-value threshold of 10^-6^, an alignment coverage requirement, and the requirement that the alignment was to a gene with assigned function. We discuss each in more detail below.

The E-value of an alignment A is the expected number of alignments of the query that would score as well as A by random chance alone, and is dependent on the length of the query and the size of the database searched against [15]. The probability of finding an alignment that scores as well as A (the P-value) is related to the E-value by the equation *P =* 1 - exp(−*E*) [7]. For the E-value 10^-6^, the P-value is also 10^-6^, which means the expected number of our 350,000 predicted genes that would align to the database of proteins by chance alone should be less than 1, according to the model used to calculate BLAST E-values. Of the 127,000 possible missed genes that had a BLAST alignment, only 78,000 had an alignment that passed through this filter.

However, many of the proteins in our database are homologous to many others, and so the database is not made of independent sequences. It is unclear as to what degree this would affect the expected number of false homologous pairs. For this reason, as well as the presence of pseudogenes, we further filter our set of alignments by requiring each alignment to cover at least 80% of the subject gene. This filter reduced our set of possible missed genes to a total of 52,605 candidate missed genes.

Finally, even if a predicted gene were an exact amino acid match to a gene in RefSeq, there is the possibility that the RefSeq gene is simply an incorrect computational prediction. The RefSeq database has many hypothetical proteins, and it is likely that a significant fraction of such genes are not true genes. Those genes that have an assigned function have such an assignment due to either an experiment verifying that function or high sequence similarity to an experimentally-confirmed gene. This means that those genes with assigned function should have a higher probability of being true genes than those without.

Therefore, we divide our remaining candidate missed genes into two groups: those with sequence similarity to a protein without the string “hypothetical” in its description (or any of several common spellings of hypothetical), designated *named missed genes*; and those with sequence similarity only to hypothetical proteins, which we designated *hypothetical missed genes*. Our analysis yielded 13,602 named missed genes and 39,003 hypothetical missed genes that we further analyzed using COMBREX.

### COMBREX

COMBREX (Computational Bridges to Experiments) is an NIH funded effort to bring computational and experimental biologists together. It serves as a clearinghouse for computationally determined gene function predictions, prioritizes these for experimental testing, and offers grants to experimental biologists to test specific predictions [10]. COMBREX maintains a database [11] of experimentally determined and computationally predicted functions for more than 3 million microbial genes. The genes in the COMBREX database are organized into functionally linked gene groups. In the default scheme, the genes are grouped into sequence-similar and likely isofunctional groups, as defined by the NCBI Protein Clusters Database [16].

COMBREX is the first functional database that attempts to provide fully traceable annotation, where predictions are traced (whenever possible) to the experimentally determined evidence. For many genes COMBREX provides a link to the nearest gene with experimentally determined function, as determined by both BLAST similarity score and shared domain composition.

In addition, COMBREX also provides information about documented phenotypes associated with each gene. Currently, this phenotype data consists of antibiotic resistance, antibiotic sensitivity, and candidate gene essentiality. Antibiotic resistance genes, obtained from Antibiotic Resistance Genes Database [17], confer resistance to one or more antibiotics through several mechanisms. Antibiotic sensitivity genes are genes that when lost, confer increased sensitivity to antibiotics [18]. The essential genes are identified, by multiple sources, as being essential for growth or viability in one or more organisms (a complete list of the organisms and sources can be found at the COMBREX web server).

### COMBREX analysis

Approximately 75% of the total missed gene set is comprised of hypothetical missed genes, for which the annotation of related proteins in GenBank provides no useful functional information. However, we were able to provide additional information for many of these hypothetical missed genes with COMBREX, by utilizing the ComBlast annotation pipeline, which we describe below.

Based on the data stored in COMBREX, ComBlast associates each missed gene with various types of important evidence or data. Consider a case of a hypothetical gene prediction identified in two closely related strains using the same gene prediction software. Clearly, this prediction could be a false positive resulting from a roughly similar k-mer distribution in the predicted region. Our confidence in the prediction grows if the same prediction is found in a large number of organisms suggesting this gene is conserved. The confidence also grows if one of the homologs has been explored experimentally. This principle is deployed systematically throughout our study. The evidence associated with each gene includes any one of the following attributes: conservation of the gene, evidence of experimentally validated function or predicted molecular function, existing 3D structures or protein domains, protein purification status, EC numbers, and gene phenotype. A missed gene is associated with specific evidence if it shares significant sequence similarity with individual genes with this type of information, or if it is assigned to one or more protein clusters in COMBREX (with at least one gene in the cluster having the required information). For the purpose of this paper, missed gene A shares significant sequence similarity with COMBREX gene B if the sequence alignment of A against B covers at least 80% of the length of B and has BLAST E-value less than 10^-5^. If gene B contains any of the above mentioned evidence, they are assigned to gene A. Gene A can also be assigned to protein cluster C if A shares significant sequence similarity with at least 100 members in that cluster (for big clusters) or all members in the cluster (for smaller clusters), or if A shares significant sequence similarity with members of only one cluster.

The information from COMBREX is used to assign each missed gene to a COMBREX support level. A missed gene is assigned to the *strong* COMBREX support level for being a true functional gene if the gene is found to be conserved in multiple organisms. We define a gene as conserved in multiple organisms if it is assigned to a cluster with more than 50 members coming from at least 2 different phyla or if it shares significant sequence similarity to more than 50 COMBREX genes. Additionally, a missed gene is assigned to the strong COMBREX support level if it has significant similarity to one or more genes associated with at least one of the following types of information: experimentally validated function with evidence, known 3D structure, presence of purified protein, protein domain, or EC number.

A missed gene not assigned to the strong support level is assigned to the *fair* COMBREX support level if it satisfies one or more of the following conditions: if it is assigned to at least one cluster having a computational prediction for its genes, if it is assigned to at least one cluster containing 5 or more members, or if it shares significant sequence similarity to 10–50 COMBREX genes. The remainder of the named missed genes that do not match the strong or fair criteria, are assigned the *weak* COMBREX support level, since they still have significant sequence similarity to genes with meaningful functional annotation. Other hypothetical missed genes that do not match the strong or fair criteria are labeled as having the *insufficient* COMBREX support level.

### Spurious gene family analysis

Along with our use of ComBlast to examine the support available for our candidate missed genes, we used AntiFam [19] to examine our candidate missed genes to determine if there is any evidence that they are not true genes. AntiFam is a database of hidden Markov models (HMMs) that represent families of genes that have been incorrectly annotated in the past frequently. In this study, we use AntiFam 2.0, which contains 47 HMMs representing families that include genes from ORFs that overlap known rRNAs, tRNAs, and other genomic features.

## Results and discussion

### ComBlast results

By the end of our search for candidate missed genes, we found 13,602 named missed genes with significant sequence similarity to a known gene with non-hypothetical annotation, and 39,003 hypothetical missed genes with homology only to hypothetical proteins. 13,307 of the named missed genes and 36,127 of the hypothetical missed genes had significant sequence similarity to COMBREX genes, and could be further analyzed using COMBREX.

**Figure 2:**
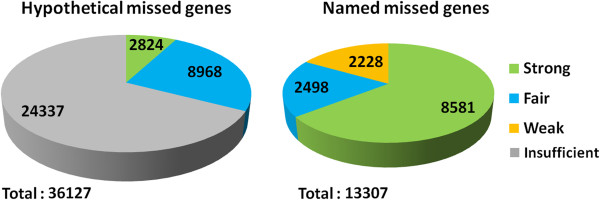
**Assignment of COMBREX support levels to the hypothetical/named missed genes using ComBlast.** For each missed gene we assign a COMBREX support level based on sequence homology and assignment to gene clusters in COMBREX. A missed gene has the *strong* COMBREX support level of being a true protein coding gene if it is conserved or associated with at least one of the following information: possessing experimentally validated function, known 3D structure, purified protein, protein domain or EC number. It has the *fair* COMBREX support level if it has a sufficient number of homologs or is associated with a predicted function. The other named missed genes, which were confirmed by sequence homology to at least one gene with non hypothetical protein annotation, have a *weak* COMBREX support level. The rest of the hypothetical genes have *insufficient* evidence and thus they are not counted in the statistics of missed genes in this paper. See the text for more detailed description of the different levels.

We started by using COMBREX to assign a confidence level to the 13,602 named missed genes. Using ComBlast, the annotation pipeline in COMBREX, we assigned 63% of the named missed genes to the *strong* COMBREX support level and 18% to the *fair* level (Figure 2). In addition, while taking into account the hypothetical missed genes, we were able to double the number of likely genes that have at least the *fair* COMBREX support level for being protein-coding genes. In total, we can assign the *fair* COMBREX support level to another 11,792 genes from the hypothetical missed gene set, which illustrates the limitations associated with sequence homology based prediction methods (Figure 2). 2,824 of the hypothetical missed genes are assigned to the *strong* COMBREX support level (for examples see Table 1), and more than 3,000 genes also have some information through functional predictions of other genes.

**Table 1:** **Examples of hypothetical missed genes that are associated with thestrongCOMBREX support level**

**Gene**	**Reasons for association with the strong support level**
AE014295_orf00919 from *Bifidobacterium longum* NCC2705	Assigned to the NCBI curated cluster PRK11770. The ORF has 213 significant sequence homologs (BLAST E-values range between 1e-58 to 3e-09) in the cluster. The cluster contains 218 genes from 125 species belonging to 6 different phyla. It has a conserved domain along with a few cloned and purified members.
CP002334_orf00644 from *Helicobacter pylori* Lithuania75	Assigned to the NCBI cluster CLSK496073. All other members of the cluster are hypothetical proteins (NCBI annotation), but COMBREX identified 3 experimentally validated genes within the cluster.
AE017354_orf01466 from *Legionella pneumophila*	Has significant sequence similarity (BLAST E-Value 1e-09) to a gene from *Aeromonas hydrophila* that is included in the gold-standard database in COMBREX (a novel set of genes with experimentally validated molecular function).
BA000023_orf01717 from *Sulfolobus sokodaii* str. 7	Has significant sequence similarity (BLAST E-Value 2e-21) to a protein from *Sulfolobus solfataricus* with NCBI annotation as hypothetical protein. However, in COMBREX this gene was identified as having a known 3D structure (PDB code: 2JTM).

**Figure 3:**
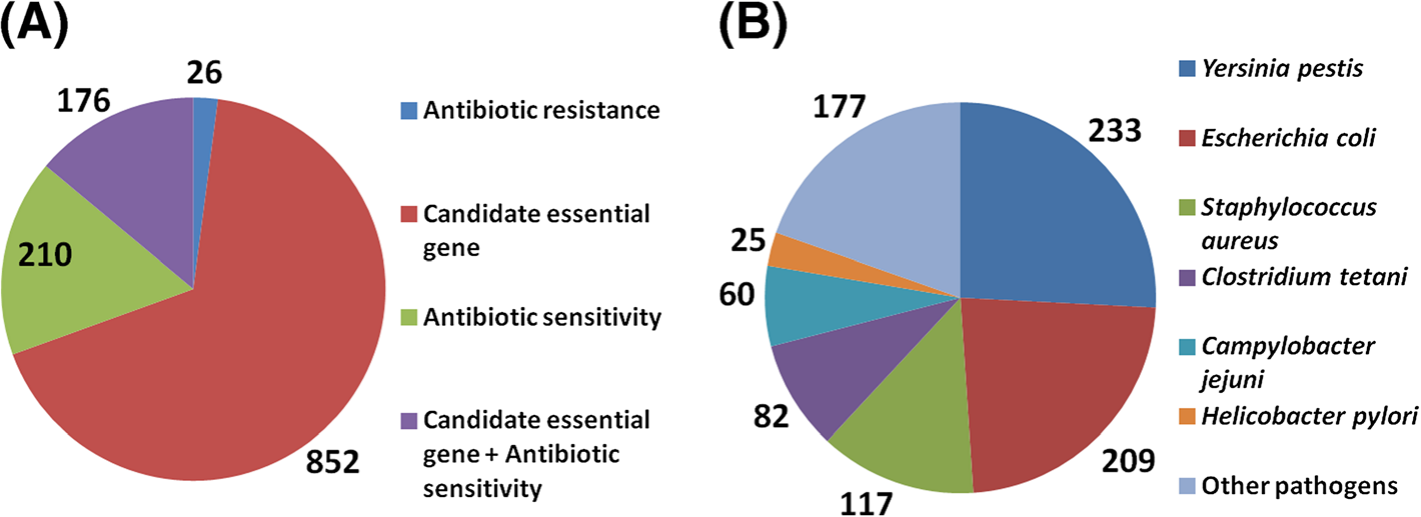
**Missed genes that can be associated with COMBREX phenotype data: (A) Phenotype data distribution.** Some of the missed genes can be associated with phenotype data using the novel COMBREX resource. A gene is associated with a specific phenotype if it has significant sequence similarity (see Methods) to a gene in COMBREX with the phenotype or if it is assigned to a cluster containing a gene with the phenotype. (**B**) Potential drug target genes (see text for details) with their distribution in pathogenic organisms (identified only to the species level). The largest portion of these genes belongs to the *Enterobacteriaceae* family of pathogens (*Yersinia*, *E. coli,* etc.) as seen in the figure. This phenotype information is pooled together using ComBlast. The full list of these genes is available online [13].

We also used COMBREX phenotype data to identify potentially important (scientifically or clinically) missed genes. We could associate 1,264 missed genes with phenotype data stored uniquely in COMBREX (Figure 3A and Table 2). Even genes that could not be associated with any meaningful functional evidence through BLAST-based analysis have been shown to contain interesting phenotype information; we found 46 such cases (see example 3 in the last column of Table 2). In our set of missed genes, we were able to find candidates that could be associated with three phenotypes: 26 could be associated with antibiotic resistance, 210 with antibiotic sensitivity, 852 with candidate essential genes and an additional 176 genes that could be associated both with candidate essential genes and antibiotic sensitivity.

**Table 2:** Examples of missed genes associated with COMBREX phenotype data

**Assigned Name**	CP001172_orf00556	CP000948_orf05287	CP002071_orf01199
**Species containing missed gene**	*Acinetobacter baumannii* AB307-0294	*Escherichia coli* K-12 substr. DH10B	*Helicobacter pylori* Sat464
**Species containing homologous gene**	*Escherichia coli* ED1a	*Escherichia coli* K-12 substr. MG1655	*Helicobacter pylori* 26695
**Name of homologous gene**	dimethyladenosine transferase	adenylate cyclase (EC 4.6.1.1)	hypothetical protein
**Blast E-value**	9e-75	0.0	1e-176
**Combrex phenotype associations**	antibiotic resistance class KsgA	picin sensitivity and triclosan sensitivity	candidate essential gene

Three representative examples of missed genes that are associated with COMBREX phenotype data: “antibiotic resistance”, “antibiotic sensitivity”, or “candidate essential gene”. Note that CP002071_orf01199 is a hypothetical missed gene with BLAST similarity only to hypothetical proteins.

Along with providing functional and phenotype related information to the missed genes, COMBREX also tries to identify which of these genes might be medically relevant. These genes may be potential drug targets in the future. To identify such cases, we considered 3 different criteria that the missed genes have to satisfy: (i) the missed gene should belong to a pathogenic organism (according to The Microbial Rosetta Stone Database of Pathogens [20]); (ii) the missed gene should be assigned to a protein cluster with at least 50 members (based on ComBlast results); and (iii) the missed gene should possess significant sequence similarity to any essential gene within COMBREX (based on ComBlast results). By this method, we found 359 missed genes, belonging to 88 different pathogenic organisms, that satisfied all the above criteria and thus might be interesting drug targets (Figure 3B). The full list of those genes is also available online [13].

### Spurious gene family analysis

To identify spurious genes in our set of candidate missed genes, the AntiFam database of HMMs was compared against both our named missed genes and hypothetical missed genes. 8 of the 13614 (0.06%) named missed genes, and 141 of the 39003 (0.36%) hypothetical missed genes were labeled as spurious according to AntiFam. We also show the number of spurious missed genes as divided by COMBREX support level in Table 3.

**Table 3:** Spurious genes found within the various subsets of candidate missed genes

**COMBREX Support Level**	**Named Missed Genes**	**Hypothetical Missed Genes**
**Strong**	8/8581 (0.09%)	51/2824 (1.81%)
**Fair**	0/2498 (0%)	46/8968 (0.51%)
**Weak**	0/2228 (0%)	0/0 (0%)
**Insufficient**	0/0 (0%)	44/24437 (0.18%)
**Totals**	**8/13307 (0.06%)**	**141/36127 (0.39%)**

The number of spurious genes and number of total genes within each combination of COMBREX support level (strong, fair, weak, and insufficient) and missed gene category (named and hypothetical) is given, as found by running candidate missed genes against the AntiFam database. This table includes only the candidate missed genes that could be analyzed by ComBlast.

The low number of genes in our named missed genes set that were labeled spurious is encouraging, as we had hoped demanding a functional assignment would result in few false positives. As may be expected, the percentage of genes in the hypothetical missed genes set that are spurious is higher than the named missed genes set, due to the lack of a functional assignment in the homologous genes used to add genes to the hypothetical set.

Five of the spurious genes (all belonging to *Haemophilus influenzae* F3031) appeared in our named missed genes set due to a single gene annotated in the RefSeq annotation of *H. influenzae* F3031 (HIBPF15861, described as “cell wall-associated hydrolase”) that AntiFam indicates is from a region that is antisense to 23S rRNA; a sixth spurious gene was due to a homologous annotated gene in *Lactobacillus crispatus* ST1. The other two spurious genes in this set, called translations of CRISPR regions by AntiFam, are homologs to two genes in *Syntrophus aciditrophicus* SB that were annotated as a “putative cytoplasmic protein” at the time we obtained our set of annotated genes in RefSeq, but now are no longer in the RefSeq record.

We also examined the 51 hypothetical missed genes that were assigned to the strong COMBREX support level, yet were labeled as spurious by AntiFam. One of these genes was a translation of a tRNA, one was contained within a repeat in the *Vibrio* superintegron, and six were contained within the insertion sequence ISlin1. The remaining 43 were labeled as translations of CRISPR regions, with 41 of those being part of a family described by a single HMM. Analysis of these 41 genes revealed that their entry into our candidate missed genes set was, in all but 3 cases, due to homologous genes that existed in RefSeq at the time we generated the set but that are no longer in the RefSeq database. In addition, all 46 of the spurious hypothetical missed genes assigned to the fair COMBREX support level were translations of antisense rRNA regions.

In summary, many of the spurious genes that we found in our set of candidate missed genes appear to have been introduced into our set by the existence of genes in a slightly outdated version of RefSeq (that have since been removed). There are also many spurious genes that overlapped rRNAs, indicating that the original annotators missed rRNA genes, as our pipeline excluded ORFs that overlapped annotated rRNAs. These two large groups of spurious missed genes, although less than one percent of our total set of missed genes, indicate two other problems with existing annotations that do not involve missing protein-coding genes.

### Missed genes analysis

In addition to finding missed genes, we conducted further analysis looking for patterns in the data that might explain why these genes were missed. Note that our analysis here is limited to the genes found via our pipeline, and does not attempt to evaluate the annotations beyond analyzing the missed genes we found.

We first checked if the center performing the annotation influenced the number of missed genes in each annotation. The majority of gene annotations were done by 4 major organizations: the Department of Energy Joint Genome Institute (JGI), The Institute for Genomic Research (TIGR), the J. Craig Venter Institute (JCVI), and the Sanger Institute. These four institutes were responsible for over half of all the annotations that we reviewed in this study. The other centers that provided genome annotations were typically smaller and only did a few genome annotations each.

In analyzing the number of missed genes by each center, we focused solely on analyzing the number of named missed genes, as we had good confidence that most of the named missed genes were true missed genes. When comparing the number of named missed genes from each of the four major centers with the number of named missed genes from the other smaller centers, we found that a higher relative proportion of named missed genes came from smaller centers, suggesting that a lack of adequate resources or experience may have contributed to the higher error rate. Reasons for missing genes might also include using a less sensitive gene finder or gene annotation pipeline.

Overall, for the four major institutes, we found between 0.71 to 3.92 named missed genes for each Mbp of sequence annotated, while for the other smaller centers, the average number of named missed genes found per Mbp was 4.48. We also computed the percent of named missed genes found versus the number of originally annotated genes. Here we found that the four major institutes missed between 0.08% and 0.43% of the genes, while the other centers missed on average 0.48% of the genes. The exact numbers of named missed genes for each of the major centers compared to other centers are provided in Table 4 along with some other statistics.

**Table 4:** Results of named missed genes analysis

**Center**	**JGI**	**TIGR**	**JCVI**	**Sanger**	**Others**	**Total**
**Chromosomes annotated**	563	95	68	67	781	1574
**Named missed genes**	1463	852	190	892	10205	13602
**Annotated genes**	1830805	254484	179284	205667	2105188	4575428
**Average named missed genes per chromosome**	2.60	8.97	2.79	13.31	13.07	8.64
**Percent named missed genes vs. annotated genes**	0.08%	0.33%	0.11%	0.43%	0.48%	0.30%
**Total chromosome length (Mbp)**	2058.6	273.9	190.1	227.7	2276.6	5026.9
**Named missed genes per Mbp**	0.71	3.11	1.00	3.92	4.48	2.71

The statistics here only count named missed genes and do not include hypothetical missed genes, as the lack of an association with a known function makes it more difficult to determine if a potential gene is a true gene.

**Figure 4:**
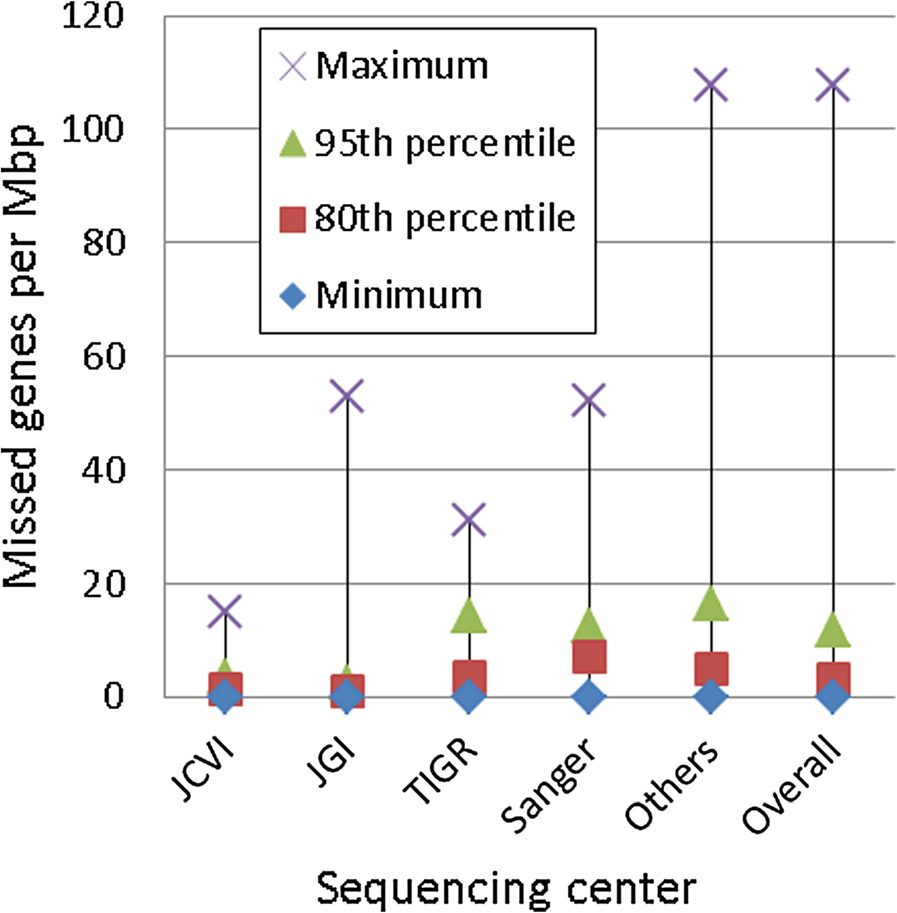
**Missed gene rate distributions per center.** For each of the four major centers, as well as the other centers as a group, a representation of the distribution of the missed gene rates for the centers is shown. For an individual center, all chromosomes annotated by that center had their missed gene rates calculated, and the 80^th^ and 95^th^ percentiles are displayed along with the minimum and maximum missed gene rates.

To examine the distribution of missed genes further, we divided the 1,574 annotations into two groups, with one group containing annotations from the four major centers, and the other group containing all other annotations; each group was then ranked by the number of missed genes per Mbp. By plotting each annotation’s rank within its group against the annotation’s number of missed genes per Mbp (Additional file 1: Figure S1), a clear visual distinction between the two groups is evident. A more detailed examination of those annotations with at least 10 missed genes per Mbp reveals that of the 97 such annotations, 79 (81%) were performed by centers other than the four major institutes. Although the major centers do have some annotations that are missing many genes, and some smaller centers miss none or very few genes, clearly the general trend is that the major centers miss fewer genes than the smaller centers. This trend is further confirmed when we examine the relationship between the missed gene rate of a center and the number of annotations the center has performed (Additional file 1: Figure S2), as well as view the distribution of missed gene rates on a per-chromosome basis within the sets of annotations performed by a particular center (Figure 4).

Another observation from our analysis of the set of named missed genes is that many of them are short genes, under 300 bp in length. Examining the shortest annotated gene for each chromosome reveals that some of the annotators likely used a minimum gene length higher than the 110 bp cutoff we used, which may account for some of these short genes being missed. In fact, for the annotations of two strains of *Yersinia pestis*, Z176003 and D182038, all 200+ missed genes were under 300 bp, while the minimum annotated gene length in the original annotations was 300 bp. In these cases, it is clear that the minimum gene length setting was the primary cause of these genes being missed.

**Figure 5:**
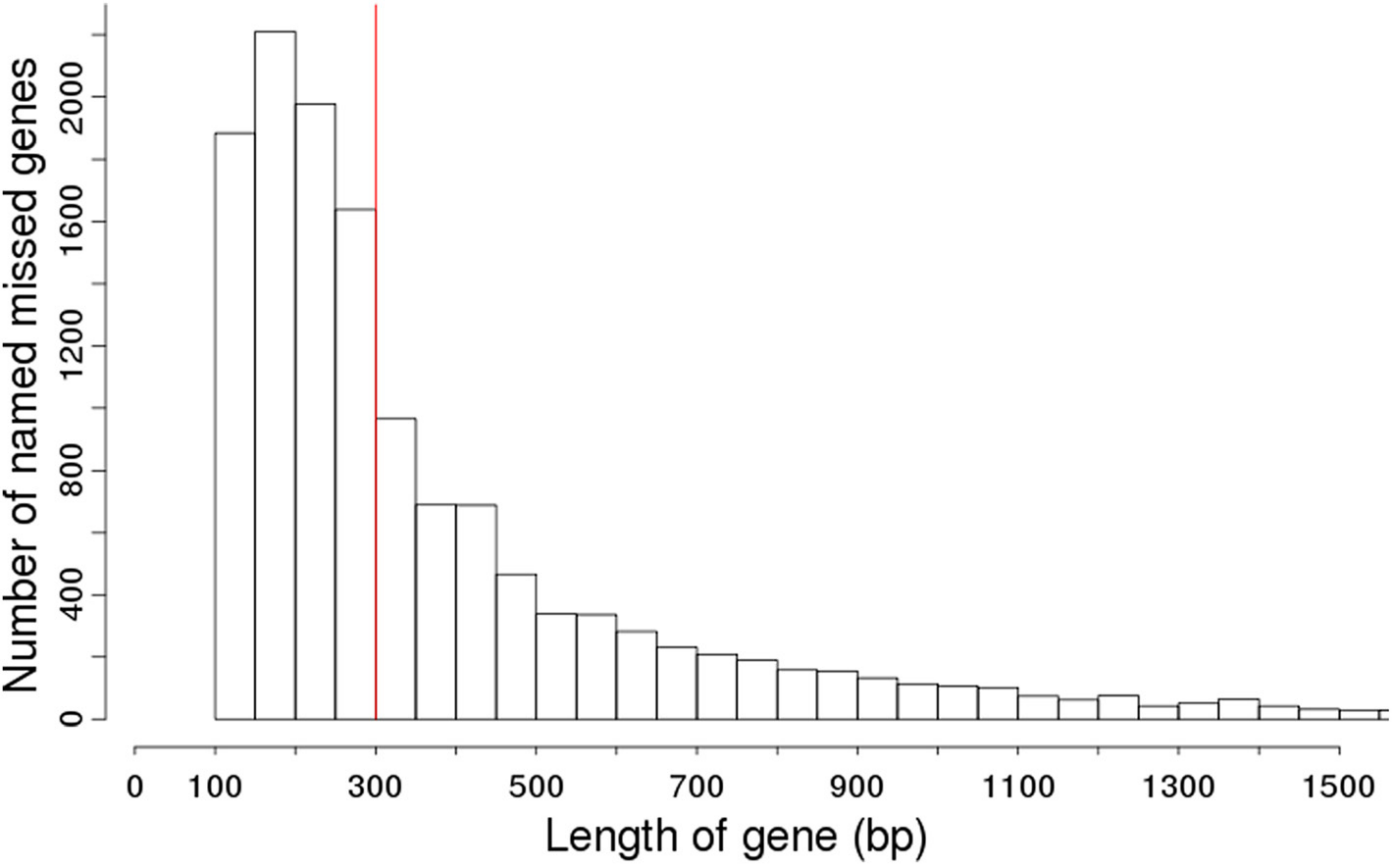
**A histogram of the lengths of 13,602 named missed genes found in 1,574 prokaryotic chromosomes.** Of the 13,602 named missed genes found in our study, 7,627 were less than 300 bp in length, indicating a strong tendency on the part of some annotators to omit shorter genes from genome annotation.

In our analysis, we used a minimum gene length of 110 bp when running Glimmer3 to find missed genes. This minimum length was used as it is the default length supplied by Glimmer3’s g3-iterated.csh script. It appears that based on the smallest annotated gene in each chromosome, some annotators use a higher minimum than 110 bp, which may result in missing shorter genes. Although short genes were not the sole cause of missed genes, we found that about 60% of the named missed genes were genes of length between 110 bp and 300 bp. A full histogram of the lengths of the missed genes we found is shown in Figure 5, and we can see that many of the genes have length less than 300 bp.

In addition to checking the distribution of gene lengths, we also checked to see if the annotations with the highest missed genes were all from several years ago, or if there were still high numbers of genes being missed in recent years. In general, it was difficult to know for certain when each annotation was done, as many annotations did not have publications associated with them, and for the ones with publications, there was still some chance that the annotation was done many years before the publication. However, we did find a number of missed genes in annotations associated with publications from the past five years. There was one particular example where the annotation of *Lactobacillus fermentum* CECT 5716 (accession CP002033) was described in a 2010 publication [21], yet the annotation still had over 100 missing genes per Mbp. This example had only 29 out of 224 missed genes under 300 bp in length, indicating that recent gene annotations may still miss many genes that are not short. One last factor we checked was to see if the missed gene rate of an annotation was influenced by the GC content of the genome, but we did not see any noticeable correlations (data not shown).

We further examined the ten genomes that had the highest named missed gene rates; these genomes are listed in Table 5. Of these ten, 8 were annotated by smaller centers, with only one annotated by JGI (*Bacillus thurengiensis*) and one by the Sanger Institute (*Streptococcus pneumoniae*). The presence of *E. coli*, *Y. pestis*, and *B. thurengiensis* genomes on this list is especially surprising, given the high number of closely-related genomes that have been sequenced and annotated, which should have made annotating these genomes easier.

**Table 5:** The ten chromosomes with the highest named missed gene rates

**Genome**	**Accession #**	**Genome size (Mbp)**	**Missed genes per Mbp**	**Total missed genes**	**% Missed genes <= 300 bp**	**Shortest annotated gene (bp)**
*N. gonorrhoeae* FA 1090	AE004969	2.15	107.7	232	35.3%	108
*L. fermentum* CECT 5716	CP002033	2.10	106.6	224	12.9%	189
*S. glossinidius* str. ‘morsitans’	AP008232	4.17	89.7	374	25.4%	99
*E. coli* APEC 01	CP000468	5.08	62.0	315	69.8%	240
*C. tetani* E88	AE015927	2.80	55.7	156	87.8%	303
*B. thurengiensis* str. Al Hakam	CP000485	5.26	52.9	278	61.5%	138
*S. pneumoniae* INV104	FQ312030	2.14	52.3	112	50.0%	99
*Y. pestis* Z176003	CP001593	4.55	50.9	232	100.0%	300
*Y. pestis* D106004	CP001585	4.64	50.9	236	99.6%	300
*Y. pestis* D182038	CP001589	4.63	50.8	235	100.0%	300

We were able to find information regarding the gene finding programs used for 7 of these ten annotations; the annotations for *Neisseria gonorrhoeae *[22], *L. fermentum *[21], and *S. pneumoniae *[23] lacked an accompanying publication detailing the annotation methods. Glimmer version 3 was used in the annotation of the three *Y. pestis* genomes [24] in Table 5. Three other annotations (*Sodalis glossinidius*, *Clostridium tetani*, and *B. thurengiensis)* used Glimmer version 2 as part of their annotation [25-27]. In addition, GenomeGambler 1.51 [28] was used to find genes in the annotation for *S. glossinidius*[25], although it is unclear how the results from GenomeGambler and Glimmer were combined. The annotation of *E. coli* used the GeneQuest program sold by DNASTAR for finding genes [29]. Looking at the shortest annotated gene in these ten annotations reveals the likely use of a high minimum gene length in the process of annotation. Five annotations have no genes less than 240 bp in length, and in each of these five annotations, the majority of missed genes were less than 300 bp in length. This appears to indicate that in these cases, the use of a high minimum gene length was the primary cause of these annotations’ high missed gene rates.

In the remaining five cases, the cause of the high rates of missed genes is less clear, largely due to a lack of information about the annotation methods used. For only two of these remaining genomes do we have a description of the gene finders used to perform the annotation, and for *S. glossinidius*, two gene finders’ results were used in an ambiguous manner.

We also wanted to determine if the genes missing from the 10 GenBank annotations listed in Table 5 were also missing from their corresponding RefSeq annotations. Although RefSeq does contain manually curated genomes for some organisms, many annotations for bacterial genomes are listed as “provisional”, meaning that they have not yet undergone final review by NCBI staff. As many RefSeq annotations for prokaryotic genomes are largely based on the genomes’ GenBank annotations when provided [14], the RefSeq annotations may still be missing large numbers of genes. Our comparison between the two sets of 10 annotations, detailed in Table 6, revealed that only 13 genes were unique to RefSeq annotations, while 43 genes were unique to GenBank annotations. Of the 13 genes unique to RefSeq annotations, 11 were in our set of named missed genes.

**Table 6:** Comparison of GenBank and RefSeq annotations for the ten chromosomes with the highest named missed gene rates

**Genome**	**GenBank annotation**		**RefSeq annotation**		**Common genes**	
	**# Genes**	**# Unique Genes**	**# Genes**	**# Unique Genes**	**# Genes**	**# 5′ changes**
*N. gonorrhoeae* FA 1090	2002	0	2002	0	2002	9
*L. fermentum* CECT 5716	1051	0	1051	0	1051	0
*S. glossinidius* str. ‘morsitans’	2432	0	2432	0	2432	13
*E. coli* APEC 01	4467	39	4430	2	4428	72
*C. tetani* E88	2373	0	2380	7	2373	21
*B. thurengiensis* str. Al Hakam	4736	0	4736	0	4736	33
*S. pneumoniae* INV104	1824	4	1820	0	1820	0
*Y. pestis* Z176003	3542	0	3546	4	3542	0
*Y. pestis* D106004	3629	0	3629	0	3629	0
*Y. pestis* D182038	3620	0	3620	0	3620	0

Genes not contained in both annotations (as determined by stop codon position) for a given genome are considered “unique”, while those contained in both are “common”. For common genes, “# 5’ changes” indicates the number of genes that have differing start codon annotations.

## Conclusions

In this study, we found and made publicly available a substantial number of genes missed in the annotations of prokaryotic genomes in GenBank. Through analysis on 1,474 completely sequenced prokaryotic genomes, we found 13,602 genes missed that had significant amino acid sequence similarity to named (non-hypothetical) genes in NCBI’s RefSeq database. We also found 39,003 missed genes which had significant sequence similarity only to genes annotated as hypothetical protein, and among those genes, we found 11,792 candidate ORFs with at least some evidence from COMBREX supporting that they are genuine missed genes. The fact that many of the hypothetical genes can be associated with evidence from known genes highlights the need for taking into account more information than just the gene description as is commonly done by many who use only BLAST. COMBREX addresses this need by providing a wider variety of information coming from many different sources. Given the large number of probable missed genes found by our method, we recommend it as an addition to bacterial annotation pipelines. Although we used Glimmer, we note other high-sensitivity gene finders, or combination of such gene finders, may also be suitable for improving gene annotations.

We found the major centers responsible for genome annotation generally had very few missed genes, and their annotations missed fewer genes on average than annotations from smaller centers. Although some smaller centers did consistently produce gene annotations with low numbers of missed genes, we found that the majority of gene annotations with a high rate of missed genes were from the smaller centers, which may have less experience in gene annotation.

We also found many short missed genes, suggesting that annotators selected a minimum gene length considerably higher than Glimmer’s default of 110 bp. Use of such a high minimum length is the likely reason behind the lack of annotation of a large fraction of the missed genes we found. Besides the common problem of missing short genes, it was difficult to determine the reasons for missing the longer genes, as the methods of annotation were not always detailed in GenBank or in the annotations’ associated publications. A survey of the genomes that had the 10 highest rates of missed genes showed a variety of annotation pipelines and software, indicating that there are several different approaches to annotation. As these approaches almost certainly differ in terms of sensitivity, those seeking to perform annotation should take care to ascertain the quality of their chosen methods.

We were also able to identify several genomes without any annotations present, and draft genomes, which appeared on a list of complete genomes on the NCBI Entrez Genome Project website. The presence of such genomes on a list purported to contain “complete genomes” should serve as a reminder to researchers that the data in our public archives is not always 100% accurate. These inconsistencies in genome annotation along with the large number of missed genes found strongly suggests the need for a common standard of best practices to be followed by gene annotation centers, and we hope this work can steer the attention of the annotation community towards this direction.

In addition to identifying many missed genes, we used COMBREX to assign phenotype information to many genes. In our phenotype analysis, we were able associate some of the missed genes with one or more phenotypes, such as antibiotic resistance, antibiotic sensitivity, and essentiality. Some of these missed genes, which are conserved in many organisms and found in potentially pathogenic organisms, could be interesting targets for the pharmaceutical community.

The cost of sequencing is decreasing at a very rapid rate suggesting that the number of organisms or clinically important strains sequenced by small laboratories without bioinformatics expertise will increase dramatically. Our results suggest a need for open access and high accuracy annotation software available to the community that combines the strengths of gene prediction programs, such as Glimmer, with information from protein databases, such as COMBREX.

## Competing interests

The authors declare that they have no competing interests.

## Authors’ contributions

DEW and HL developed and implemented the pipeline for discovering missed genes. ALM planned and directed the development and the implementation of ComBlast and the analysis of the data using COMBREX. YCC and RS made substantial contributions for the development and implementation of ComBlast and for the analyzing the data. All authors participated in design of the study and writing the manuscript. All authors read and approved the final manuscript.

## Reviewers’ comments

Reviewer 1: Dr. Daniel Haft

Wood and coauthors describe a study that seems to have two separate and complementary purposes – to showcase how standardizing on GLIMMER3 used with properly set parameters might improve gene finding for prokaryotes, and to showcase how BLAST searching vs. the COMBREX database of genes with experimental evidence allows better verification of coding region predictions than searching unfiltered databases. Unfortunately, each purpose is somewhat compromised by bundling the two tasks without benchmarking each phase of the project independently.

Because GLIMMER3 relies on analyses of k-mer frequencies in trusted sets of coding regions from a single genome, it models what is typical across a single genome. Consequently, islands present in that genome because of lateral gene transfer (LGT) are handled much more poorly than the rest of the genome. If the point is to find large numbers of genes that may have been missed, in a computationally efficient manner, where those candidates are slated to be filtered through a subsequent validation process anyway, then that the pipeline probably should use GLIMMER3 and MetaGene in combination, taking the union, and the pipeline should not filter out plasmid replicons (an area of relative weakness for GLIMMER3, which needs a large replicon to generate good statistical models).

It would be good to know from this study how severe the issue of missed genes in public databases actually is. In fact, the average number of genes picked up per archival genome submission to GenBank is small in this paper, on the order of 10 new genes for each 4000-gene genome, suggesting accuracy has actually been very good all along, other than the conspicuous exceptions noted in the paper. A previous study looked at every intergenic ORF, and found similar numbers, but that study would not have seen genes overshadowed by spurious genes and spurious extensions to genes. The real problem of missed genes may run closer to 3 % (by count, not by length, since missed genes tend to be small). The current paper uses GLIMMER3 as the only gene-finding method, and to a large extent finds genes missed by GLIMMER in earlier incarnations or run with inappropriate parameters. I recommend that the *ab initio* gene-finding phase of this study be redone, using the union of GLIMMER and MetaGene predictions instead of GLIMMER only (after which I would amend my remarks here).

Authors’ response: The use of multiple gene-finding programs, and taking the union of their results, would certainly be a worthwhile step were one seeking to build a full annotation pipeline, or even to find as many missed genes as possible; as we stated in our introduction, however, this is not what we intended with this study. We sought to provide a simple pipeline that would quantify the number of new missed genes that could be found using just a few freely available tools and resources (Glimmer3, BLAST, and RefSeq). With few exceptions, genes found by such a pipeline should simply not be missing from existing annotations, and if they are absent from the annotation, a reason for doing so should be present in the annotation itself (e.g., the annotators believe such genes to be pseudogenes). Although there are almost certainly genes that we did not find as part of this study, we believed that by focusing attention on these unannotated genes and the reasons for their absence, we can bring attention to the many various methods of annotation currently in use and the need for standardization.

While we agree with the reviewer that average sensitivity appears to be quite high, the “conspicuous exceptions” we noted are quite important, and a large part of what we aimed to highlight with this study. The lack of review given to GenBank submissions is not necessarily well-understood by the entire genomics community, and as we mention later in reply to the reviewer’s point about RefSeq, errors and omissions from a GenBank annotation can often be propagated into the respective RefSeq annotation. Bringing these errors in the public genomic records to the community’s attention is one of the main goals of our paper.

The second phase of the study attempts to demonstrate how validating predicted gene calls is improved by use of a BLAST-searchable database of proteins in which direct experimental evidence is distinguished from transitive evidence of function, and both are distinguished from sequences with no evidence of representing real genes. While it is natural to pair this part of the study with the data stream of predicted missed gene calls from the *ab initio* work with GLIMMER, it is regrettable that there is no benchmarking of the COMBLAST pipeline and COMBREX data set using more typical data than the less-than-1-percent of atypical genes found in phase 1. How does COMBREX do with the complete genome of an endosymbiont such as *Blochmannia floridanus*? How does it do with a large, GC-rich genome such as *Mycobacterium smegmatis*? The paper does not describe proteomics as a source of evidence for confirmation in COMBREX that a gene is translated into protein, so perhaps the best test is a set of proteomics-verified polypeptides from, say, *Yersinia*.

It is difficult for the reader/reviewer to evaluate whether or not there may be a problem in COMBREX – that some of the evidence in COMBREX may point precisely to a genomic region and yet not necessarily indicate the correct reading frame for that gene. Experiments based on antisense RNA, or transposon mutagenesis, do not point to a specific reading frame. The test I recommend is to select some GC-rich genome, make a test set by GLIMMER *ab initio* gene predictions, make a negative control set from all the longest ORFs that GLIMMER rejected from the genome, run both sets through the COMBLAST pipeline, and compare those results.

*Authors’ response*: *The types of analysis suggested by the reviewer are worth doing in order to evaluate the advantages of using the newly available ComBlast tool and the COMBREX database. However, this was not the focus of our work. Currently we do not consider ComBlast as a tool for discovering missed genes. However, in the context of this work, COMBREX becomes a useful resource as it provides fully traceable annotation (whenever possible) to the experimentally determined evidence. As such we use it to provide sources of evidence for some of the missed genes, which we hope will help to focus the attention of the community on that topic. We demonstrated here a simple yet important use of that type of information, which we believe should be an integral part of any annotation pipeline. It is important to make clear that we do not intend to classify the missed genes as true and false positives based only on COMBREX, since there might be many missed genes that are indeed protein-coding genes that COMBREX cannot currently associate with any experimental evidence. In this study we also used COMBREX to identify biologically interesting potential genes, which others might want to further investigate. In the future, we do plan to continue developing the abilities of ComBlast and publish a more complete study evaluating its capabilities. We hope such a study will provide some answers to the important questions pointed out by the reviewer.*

Here are a number of additional points about the paper.

One mechanism to evaluate COMBREX for spurious gene calls is to search it with a database of HMMs built to detect some of the most popular spurious gene calls – AntiFam. These models should be used to check both COMBREX genes with evidence and GLIMMER’s candidate missed genes.

Authors’ response: We thank the reviewer for bringing AntiFam to our attention. We used AntiFam to check both the named missed genes as well as the hypothetical missed genes; 8 of the 13614 (0.06%) named missed genes were found to be spurious genes, as were 141 of the 39003 (0.36%) hypothetical missed genes. We have added discussion of this to the manuscript and a new table summarizing the results.

The statement that a gene has evidence is very different from the statement that the full length of a gene has evidence. Thus, a spurious ORF from one genome could be compared to a real gene, with an improper 5’-extension, from another genome. The COMBLAST pipeline would provide evidence in support for adding the gene, and naming it, when that action might be inappropriate.

Authors’ response: Although this is possible, ComBlast does attempt to prevent such occurrences by only considering genes to be similar if the alignment between the two covers at least 80% of the gene in COMBREX. As the reviewer mentioned earlier, 5’ annotation remains a more challenging problem than gene identification, and the lower accuracy of start codon annotation compared to gene annotation should be taken into account by anyone using ComBlast, or any gene finding/annotation tool. We do not claim that every missed gene identified is necessarily correctly called as a gene and associated with a name, but only that it has good evidence to support that it is a true gene. Further analysis and checking may need to be performed to confirm each gene's validity.

The paper makes no mention that NCBI provides RefSeq versions of genomes, produced by a pipeline that is applied pretty consistently. The purpose of RefSeq is to compensate for the fact that submitted genomes are largely archival documents, not maintained with respect to functional annotation and only infrequently modified to correct previous gene-calling errors. Thus, genes missing from GenBank are not equivalent to genes missing from the accessible world of searchable protein sequences.

*Authors’ response: While annotations of genomes in RefSeq are provided by NCBI, many of these are still listed as “provisional”, meaning that they have not been manually reviewed by NCBI staff. For prokaryotic genomes, the annotation present in RefSeq is based heavily on the GenBank annotation if one is provided*[14]*. We have added a discussion on this, as well as a comparison of RefSeq and GenBank annotations for ten genomes. In summary, although genes missing from GenBank are not necessarily missing from RefSeq, a sample of missed genes from GenBank we found were also missing from RefSeq.*

Reviewer 2: Dr. Arcady Mushegian

The study by Wood et al. reports the results of re-prediction, by Glimmer3, of the open reading frames in the majority of finished bacterial genomes and identifying such of these genes that have been missed by earlier genome annotation efforts. The protein-level sequence similarity to the database proteins is used as a criterion of the reality, which is further elaborated by taking into account the experimental knowledge about the homologs of the predicted gene products. The COMBREX database, which holds and curates this knowledge, is discussed, and examples of database queries that have to do with antibiotics resistance and gene essentiality and help guide experiments are given. The study also collected statistics concerning missed genes in genome annotations. For example, the average number of missed conserved genes per annotated genome appears to be about 10, this average varies between 2 and 13 depending on the sequencing center, large sequencing centers are better than small teams - presumably, because of the more robust annotation pipelines and better staffed bioinformatics department - and genes missed by all centers tend to be on the shorter side - evidently, in part because of the arbitrary length cutoffs imposed by many genome annotation efforts.

COMBREX is a useful resource, set up in such a way as to involve the scientific community in improving annotation of bacterial gene function. This is worth highlighting. On the other hand, my perfunctory attempts to find new predictions, i.e., previously missed but now restored to being, genes by querying http://combrex.bu.edu/ failed - is there a way to do it, and should it not be provided simultaneously with the submission of a paper that talks about such genes?

*Authors’ response*: *It is one of our goals to make sure that the new set of missed genes will be accessible to the community. To insure that, we first provide a webpage on COMBREX’s database website with all the missed genes, including the ones that can be related to COMBREX genes. One can also query the COMBREX database with a specific sequence using the ComBlast server currently under development at*http://scibaydev.bu.edu/*. We plan to develop both the user interface and the tool itself so it will be as useful as possible to general users. Finally, it is our intention to include the missed genes as part of the COMBREX database. This way every missed gene will be available to queries to the database.*

A technical comment: on p. 4, we read: "For those candidate missed genes with homology only to hypothetical proteins, we needed additional information to determine if they were indeed genes." and again on p. 7: "it is likely that a significant fraction of such genes [i.e., those passing three reasonably restrictive filters, after sequence similarity has been established in the first place - AM] are not true genes". In both cases, it is not clear to me what the alternative to these ORFs being true genes may be - they could be pseudogenes of course, but the authors address this separately. Then, on p. 8, the authors suggest an alternative for the special case of very closely related strains, when the conservation of a spurious ORF can be an artifact of similar k-mer distribution (I suppose, even more trivially in this case, this could be a result of very high overall nucleotide-level sequence similarity). Right away, however, the authors remark that if an ORF is conserved in more genomes, and, better yet, in several relatively diverse evolutionary lineages, then it is most likely not spurious. But since the ORFs in question were identified by sequence similarity in the first place, one would think that a simple filter on the taxonomic closeness or even percent identity (not too high in either case) would take care of the problem? More generally, and in line with the authors' approach, it would be useful to have a rule of thumb such as "an ORF with a homolog separated by evolutionary distance X has an Y percent chance not to be spurious" - most likely, the authors already have data on hand to address this?

Authors’ response: Although such filters would very likely solve the problem, we wanted to be as sure as possible that the missed genes we considered as part of our annotation center analysis were indeed true genes. To that end, we elected to require the presence of a functional assignment to the gene, rather than attempting to discover a percent identity threshold that was “conservative enough” to give us a similar confidence in a gene’s coding nature.

p. 8 and Table 1: replace "significant homology" with "significant similarity".

Authors’ response: We have made these changes, and thank the reviewer for bringing them to our attention.

Quality of written English: Acceptable

Reviewer 3: Dr. M. Pilar Francino (nominated by Prof. David Ardell)

This work reports an interesting reanalysis of gene annotation in bacterial genomes, revealing that, although the great majority of genes are found in every genome, a large number of very likely genes have been missed overall. Many of these misses are due to overstringent cut offs in terms of minimum gene length. The analysis also reveals that large genome centers that rely on well established annotation pipelines miss fewer genes than smaller centers and individual laboratories, suggesting that bioinformatics expertise is another crucial factor in this issue. The missed genes are separated by the authors into “named” and “hypothetical” groups and further analysed using the new COMBREX database, which contains functional and phenotypic gene information that has been gathered from the experimental literature. This provides further support for the coding nature of the candidate missed genes in the “named” group and for a fraction of those in the “hypothetical” group. Moreover, a specific level of support is assigned to every gene annotation depending on the type of COMBREX information associated with it. Overall, the paper is an important attention call on the need to homogenize annotation procedures as well as a demonstration of how knowledge bases such as COMBREX can facilitate and improve gene annotation. It is extremely well written and easy to follow.

Quality of written English: Acceptable

Authors’ response: We thank the reviewer for her kind comments.

## Supplementary Material

Additional file 1 **Figures S1 and Figure S2.** Plots of prokaryotic annotations organized by rate of missed genes; and relationship between the named missed gene rate of a center and the number of annotations performed. Click here for file
