# Supplementary material for: Thousands of missed genes found in bacterial genomes and their analysis with COMBREX
Source: Biol Direct. 2012 Oct 30;7:37. doi: 10.1186/1745-6150-7-37 (PMC3534567; doi:10.1186/1745-6150-7-37)
Supplement: Additional file 1 — Figures S1 and Figure S2. Plots of prokaryotic annotations organized by rate of missed genes; and relationship between the named missed gene rate of a center and the number of annotations performed. [file 1745-6150-7-37-S1.pdf]

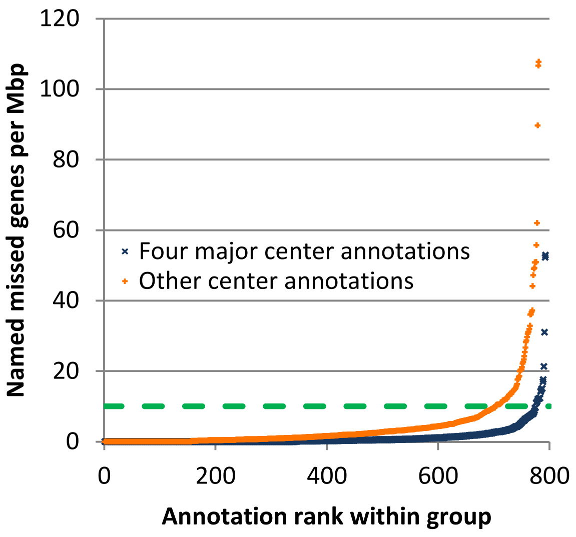

Supplementary Figure 1: Plot of prokaryotic annotations organized by rate of missed genes. Each of the 1,574 annotations studied was placed into one of two groups: the 793 annotations done by one of the four large centers (JGI, TIGR, JCVI, and the Sanger Institute), and the 781 other annotations done by the smaller centers. The annotations were then sorted for the two groups separately, in increasing order by the number of named missed genes found in the annotation per Mbp of genome sequence. The number of named missed genes per Mbp was then plotted for each annotation according to its rank within the sorted ordering of its group of annotations. The resulting plot shows the distribution of the number of named missed genes per Mbp for annotations provided by the four large centers and by all other centers; each blue 'x' represents an annotation performed by one of the four major annotation centers, while each orange '+' represents an annotation performed by one of the other smaller centers. A green dashed line marks the threshold of 10 named missed genes per Mbp. Note that since there are roughly the same number of annotations performed by the large centers as the smaller centers, the blue 'x's appearing below the orange '+'s indicate that the large centers generally produced annotations with fewer named missed genes per Mbp.

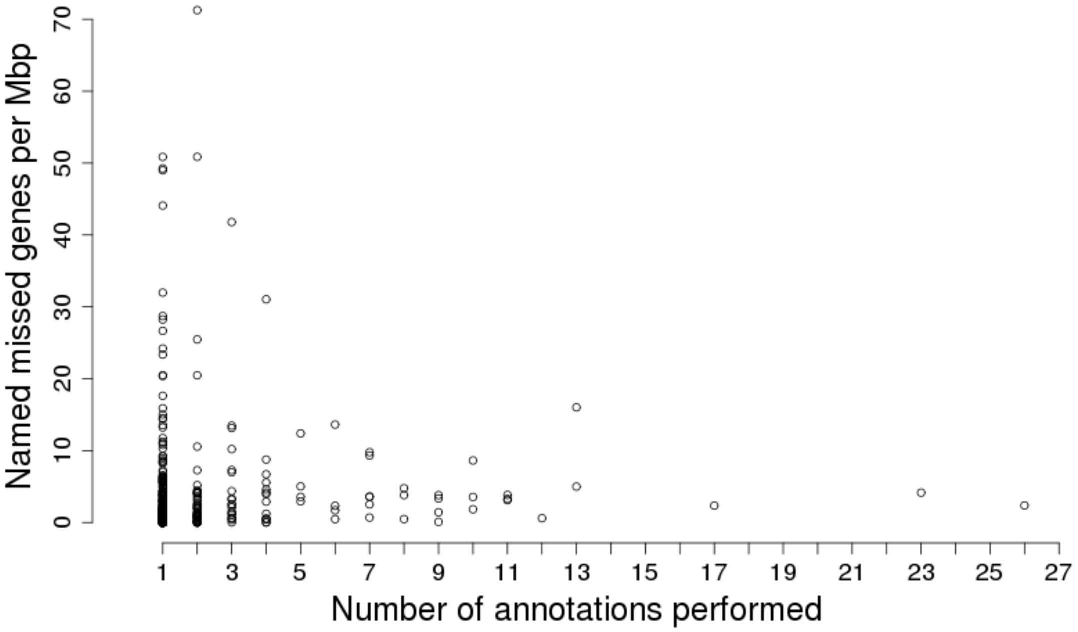

Supplementary Figure 2: Relationship between the named missed gene rate of a center and the number of annotations performed. Each point on the plot represents a single annotation center, and indicates the number of annotations the center has performed as well as the number of named missed genes per Mbp of sequence annotated by the center. The four major annotation centers (JGI, TIGR, JCVI, and Sanger) do not appear in the figure above, as they each have done over 60 annotations, but they all have less than four named missed genes per Mbp.
